# Medical Experts

**Published:** 1881-02

**Authors:** 


Medical Experts.—A trial took place recently in the Common Pleas Court of Montgomery County, which afforded an incident of some interest to the profession. It was a suit for damages alleged to have been inflicted by one woman on another while scuffling in a playful way.—Expert testimony was called as to injuries to the shoulder-joint and arm, and it was proposed that a personal examination be made of the plaintiff and an opinion given as to the condition of the parts. Dr. Reeve, of Dayton, being on the stand demurred to this and appealed to the Court, urging the injustice of a medical witness being compelled to make such examination without compensation, and declining to make it unless under direct order of the Court. Judge Elliott, in reply, fully and freely recognized the fact, that a professional man's education was his capital and ought not to be called upon without recompense. The attorneys also openly deplored the injustice of the law in such cases and admitted that a reform was much neeeded. Finally, the party interested agreed to pay a fee and the trial proceeded.

There is no doubt that Dr. Reeve's example might often be followed to advantage. The Courts would, we believe, generally recognize the injustice of the proceeding, and one so widely different from that pursued toward lawyers in similar circumstances, as was pertinently urged by Dr. Reeve.—*Cincinnati Lancet and Clinic.*

